# Photocatalytic Degradation of an Aromatic Pharmaceutical over TiO_2_: Experimental and Computational Insights into Inhibition Effects of Natural Organic Acids

**DOI:** 10.3390/molecules31050794

**Published:** 2026-02-27

**Authors:** Andrijana Bilić, Sanja J. Armaković, Stevan Armaković

**Affiliations:** 1 University of Novi Sad, Faculty of Sciences, Department of Chemistry, Biochemistry and Environmental Protection, 21000 Novi Sad, Serbia; andrijana.bilic@dh.uns.ac.rs; 2 University of Novi Sad, Faculty of Sciences, Department of Physics, 21000 Novi Sad, Serbia; stevan.armakovic@df.uns.ac.rs

**Keywords:** photocatalysis, aromatic micropollutant, competitive adsorption, noncovalent interactions, density functional theory, periodic density functional theory

## Abstract

The photocatalytic degradation of the pharmaceutical compound nadolol over TiO_2_ under UV-LED irradiation was investigated, with particular emphasis on the inhibitory effects of common low-molecular-weight organic acids. Due to its aromatic (tetralin-like) motif and multiple heteroatom-containing functional groups, nadolol serves as a representative model for aromatic micropollutants whose fate can be governed by surface competition and noncovalent interactions. While TiO_2_ showed high photocatalytic activity in ultrapure water, achieving complete nadolol degradation within 120 min, the presence of citric, oxalic, and acetic acids markedly reduced the degradation efficiency by approximately 72%, 62%, and 29%, respectively. Experimental results demonstrated that this inhibition could not be attributed solely to pH changes, indicating the contribution of additional molecular-level effects. To elucidate the underlying mechanism, molecular and periodic density functional theory (DFT) calculations were performed. The computational analysis revealed strong interactions between nadolol, organic acids, and the TiO_2_ surface, leading to competitive adsorption and partial blocking of photocatalytically active sites. These results provide mechanistic insight into the role of natural organic acids in TiO_2_-based photocatalytic systems and highlight the importance of considering real-water matrix components when designing efficient and sustainable photocatalytic water treatment processes.

## 1. Introduction

The global consumption of organic compounds is rising, driven by increased industrialization, advanced agricultural practices, and technological advancements [1]. Unfortunately, this growth is accompanied by increased organic contamination and the release of toxic waste into the environment. This threatens ecological systems worldwide, making environmental remediation an urgent priority [2]. The pharmaceutical and agricultural industries release large amounts of residues containing trace levels of pollutants, which pose significant risks to the environment and human health [3].

**Figure 1:**
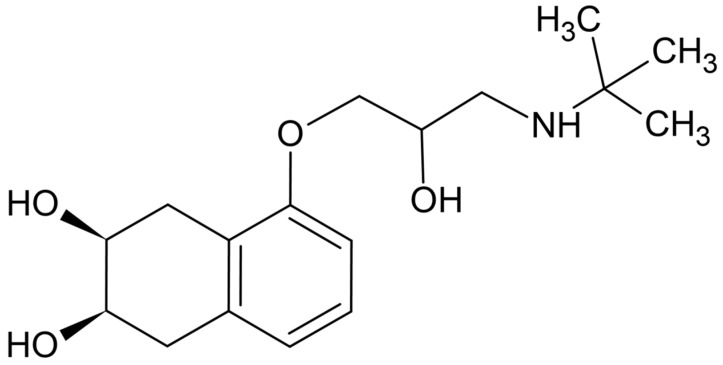
The structure of nadolol.

Polycyclic aromatic compounds (PACs) represent a broad group of environmentally relevant aromatic contaminants, including heteroatom-containing systems, whose environmental fate is strongly influenced by noncovalent interactions and surface affinity rather than by bulk solution properties alone [4,5]. Beta-blockers, prescribed for cardiovascular diseases like angina, hypertension, arrhythmias, and post-heart attack recovery, are widely used pharmaceuticals [6]. In this context, nadolol (Figure 1), although classified as a pharmaceutical compound, exhibits structural features relevant to PAC-like surface interactions, making it suitable for mechanistic studies of surface-controlled degradation processes. Nadolol is partially excreted unchanged (24.6%) via urine. However, like many pharmaceuticals, it is not effectively removed from wastewater, leading to its detection in natural water at a concentration of 0.062 µg/dm^3^ [7].

Citrus wastewaters are by-products generated during the processing of citrus fruits, with over 700 million m^3^ produced annually worldwide. These wastewaters are characterized by high levels of organic acids and a low pH, posing a threat to environmental health [8]. Citrus wastewaters contain citric, oxalic, and acetic acids, with citric acid substantially more concentrated than the others [9].

Several methods for degrading organic compounds are available, including wet oxidation, biological oxidation, electrochemical redox, advanced oxidation processes (AOPs), and combined approaches. Photocatalysis, a type of AOP, offers a promising solution using light energy to degrade pollutants into harmless byproducts. The generation of reactive species, such as hydroxyl radicals, with an oxidation potential of 2.8 eV, makes photocatalysis a robust process capable of completely mineralizing organic pollutants [10]. Among the various photocatalysts, titanium dioxide (TiO_2_) has gained significant attention due to its high photocatalytic activity, chemical stability, non-toxicity, and cost-effectiveness, making it one of the most widely used materials in photocatalysis [11]. Because of its unique properties, TiO_2_ has been utilized in a variety of applications across different fields [12,13,14].

Commercial TiO_2_ was intentionally used as a reference photocatalyst to decouple material design effects from interaction-driven inhibition phenomena and to focus on mechanistic aspects relevant to environmentally realistic aqueous systems.

In this study, the degradation of nadolol in the presence of TiO_2_ under UV-LED radiation was conducted in ultrapure water (UPW). The effect of the commonly detected organic acids in wastewater on the efficiency of photocatalytic removal of nadolol, with the addition of citric, oxalic, and acetic acids, was also investigated. The presence of these acids in aquatic environments is well-documented [9], but the current level of understanding of their effects on the stability, transformation, and degradation of micropollutants, such as nadolol, is still limited [15].

Although real wastewater represents a complex mixture of organic compounds, investigating individual organic acids is essential for understanding their specific roles and interaction mechanisms in photocatalytic systems. This approach enables clearer mechanistic insight into how different functional groups influence pollutant removal processes.

To further elucidate the structural and interaction effects governing inhibition phenomena, atomistic calculations based on semiempirical and density functional theory (DFT) methods were performed. Such computational approaches are widely applied in the study of photocatalytic and environmental systems, particularly for TiO_2_- and ZnO-based materials, with significant relevance in water treatment applications [16,17,18,19,20,21]. Both molecular and periodic DFT calculations were employed. Noncovalent interactions were analyzed using the reduced density gradient (RDG) approach, while TD-DFT simulations of UV spectra provided insight into differences in photoabsorption behavior among the investigated systems. This combination of approaches is widely employed in the analysis of various molecular properties [22,23,24,25,26,27], which is very useful for understanding the intrinsic reactive properties of molecular structures. It is also closely related to the BerchNCI protocol defined by Thomas [28,29,30,31], which is of particular interest for understanding interactions between relevant structures.

By combining experimental and computational analyses, this study provides a mechanistic understanding of inhibition mechanisms in TiO_2_-based photocatalysis and clarifies the role of natural organic acids under environmentally relevant conditions.

## 2. Results and Discussion

### 2.1. Photodegradation of Nadolol Using TiO_2_

**Figure 2:**
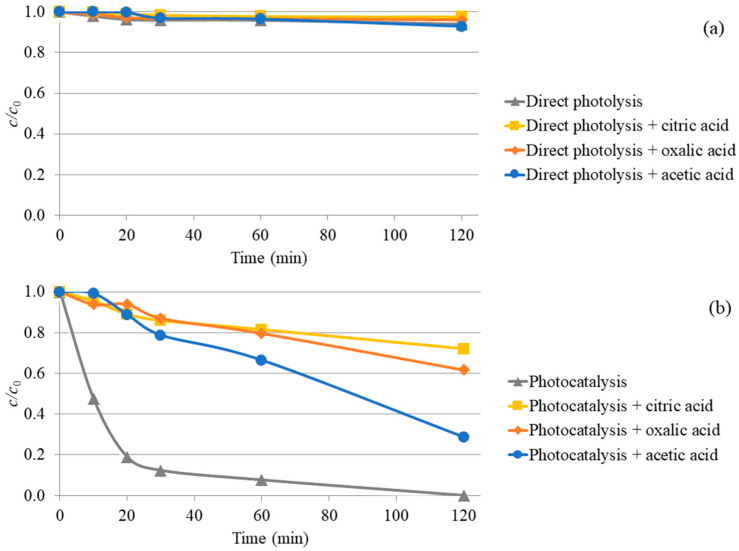
Nadolol removal (0.05 mmol/dm^3^) by (**a**) direct photolysis and (**b**) TiO_2_ (1.0 mg/cm^3^) photocatalysis in the presence of organic acids (3.00 mmol/dm^3^) under UV-LED irradiation.

The ability of TiO_2_ to remove nadolol under UV-LED irradiation in an open photochemical cell (Figure 2) was evaluated by comparison with direct photolysis under identical conditions without the catalyst. Nadolol showed high photolytic stability in UPW under UV-LED irradiation with only 6% nadolol degradation after 120 min (Figure 2a).

The influence of the three most frequently present organic acids in wastewater, citric, oxalic, and acetic acids, on the efficiency of direct photolysis of nadolol was examined.

The results of direct photolysis of nadolol in the presence of citric, oxalic, and acetic acids show that their addition did not substantially change the photostability of nadolol in UPW, which implies these acids do not act as photosensitizers under the experimental conditions (Figure 2a).

Contrary to direct photolysis, the addition of TiO_2_ nanoparticles substantially increased the efficiency of nadolol degradation, which could be expected. After 30 min of irradiation, the efficiency of photocatalytic degradation of nadolol was 88%, while nadolol was completely degraded after 120 min (Figure 2b). However, considering the complexity of real wastewater matrices, the effect of the selected organic acids on the photocatalytic activity of TiO_2_ was further investigated. The presence of citric, oxalic, and acetic acids substantially influenced the efficiency of nadolol photodegradation using TiO_2_. Specifically, the efficiency of nadolol degradation was reduced by 72%, 62%, and 29%, respectively, in the presence of citric, oxalic, and acetic acids for 120 min of irradiation (Figure 2b). With the addition of citric acid, the pH value of the nadolol solution in the presence of TiO_2_ decreased from 6.0 ± 0.1 to 2.9 ± 0.1, while it decreased to 2.4 ± 0.1 and 3.7 ± 0.1 in the presence of oxalic and acetic acid, respectively.

**Figure 3:**
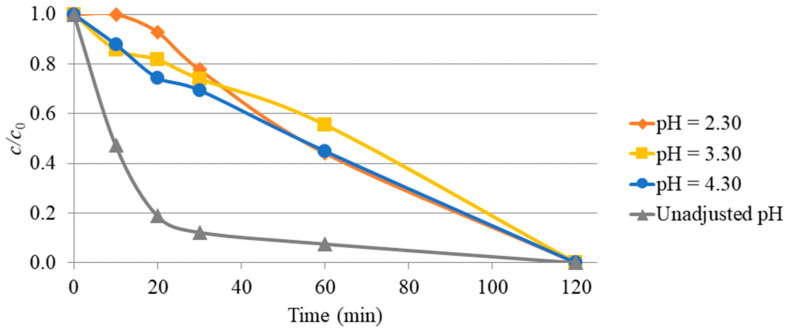
Photodegradation of nadolol (0.05 mmol/dm^3^) under UV-LED irradiation with TiO_2_ (1.0 mg/cm^3^) as photocatalyst at different pH values.

Previous studies show that the pH value has a significant effect on the photocatalytic activity of TiO_2_ and other oxide materials [32]. The efficiency of nadolol degradation using TiO_2_ was also tested at adjusted pH values of 2.4 ± 0.1, 3.4 ± 0.1, and 4.4 ± 0.1, using the addition of perchloric acid. Although the total degradation of nadolol after 120 min of irradiation remained approximately the same at all adjusted pH values, a decrease in degradation efficiency was observed (Figure 3), which indicates the sensitivity of the process to pH conditions.

Acidic conditions are known to modify the surface charge of TiO_2_ and affect hydroxyl radical generation, primarily influencing degradation kinetics without altering the final degradation outcome under the applied experimental conditions [10,25].

The results show that the reduced efficiency observed in the presence of citric, oxalic, and acetic acids cannot be attributed to pH effects alone. This indicates that intermolecular interactions and surface competition effects also contribute. Here, the term “competitive adsorption” refers to interaction and competition at the catalyst surface under photocatalytic conditions rather than to a classical adsorption equilibrium under dark conditions.

Such behavior is consistent with PAC-like systems, where removal efficiency is largely governed by surface competition and noncovalent interactions.

No significant adsorption was observed under dark conditions within the detection limits of the applied analytical method.

To clarify these effects at the molecular level, complementary molecular and periodic DFT analyses are presented in the following sections.

The obtained results were compared with literature data for TiO_2_-based systems used in the degradation of beta-blockers (Table 1). The applied TiO_2_ showed activity comparable to several previously reported systems, even without additional material modification.

### 2.2. Binding Energies

**Figure 4:**
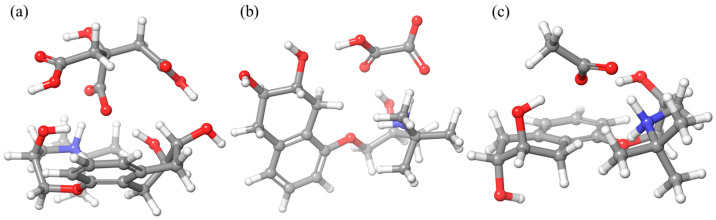
Optimized geometry of the ground state of the intermolecular system of nadolol and (**a**) citric, (**b**) oxalic, and (**c**) acetic acid. Atom color coding: carbon—grey, oxygen—red, nitrogen—blue and hydrogen—white.

For a deeper understanding of these effects, a detailed theoretical analysis of the reactivity and interaction of nadolol with the organic acids was conducted. Through geometric optimization within molecular DFT calculations, stable conformations of the intermolecular nadolol/organic acid system (Figure 4) with binding energies for each system (Table 2) were obtained.

The value of the binding energy between nadolol and an organic acid indicates the stability of the formed intermolecular system. The results showed that in the case of nadolol and citric acid, the binding energy is the strongest, which makes this system the most stable compared to the others. The above results explain the greatest decrease in nadolol degradation efficiency using TiO_2_ in the presence of citric acid (Figure 2b). The weakest binding energy was observed between nadolol and acetic acid, which is also consistent with the photocatalysis experiments.

In PAC-like systems, such trends reflect the decisive role of molecular size, functionalization, and available contact area in governing adsorption strength and environmental persistence.

**Figure 5:**
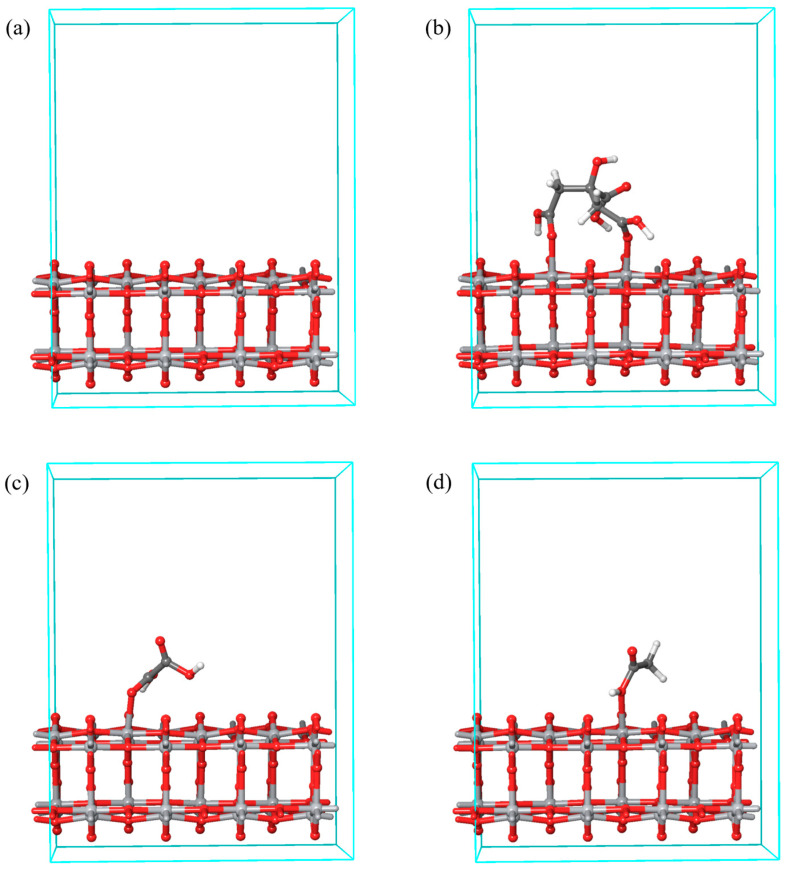
Optimized geometry of the ground state (**a**) TiO_2_ and systems (**b**) TiO_2_/citric, (**c**) TiO_2_/oxalic, and (**d**) TiO_2_/acetic acid. Atom color coding: titanium—light grey, carbon—dark grey, oxygen—red, hydrogen—white.

To better understand the difference in the efficiency of nadolol degradation using TiO_2_ in the presence of organic acids, a geometrical optimization of the TiO_2_/organic acid system was carried out using periodic DFT calculations (Figure 5). Also, the binding energies between the representative TiO_2_ anatase surface and the corresponding organic acid were calculated (Table 2).

The binding energies between TiO_2_ and organic acids decrease in the order of citric > oxalic > acetic acid. This sequence indicates how organic acids reduce the efficiency of the photocatalytic degradation of nadolol when present. Namely, citric acid, which inhibits the mentioned process in the highest percentage (Figure 2b), creates the strongest interaction with TiO_2_, which leads to easy binding to the active centers of the photocatalyst. Consequently, the number of TiO_2_ active centers where nadolol degradation can occur is reduced. On the other hand, acetic acid has the weakest interactions with nadolol and TiO_2_, so the effect of inhibiting the photocatalytic degradation of nadolol is the smallest in its presence.

The stronger inhibition by citric acid is consistent with its multidentate anchoring capability and extended hydrogen-bonding network, which promotes stronger and more persistent occupation of surface sites.

### 2.3. Noncovalent Interactions Based on Reduced Density Gradients

**Figure 6:**
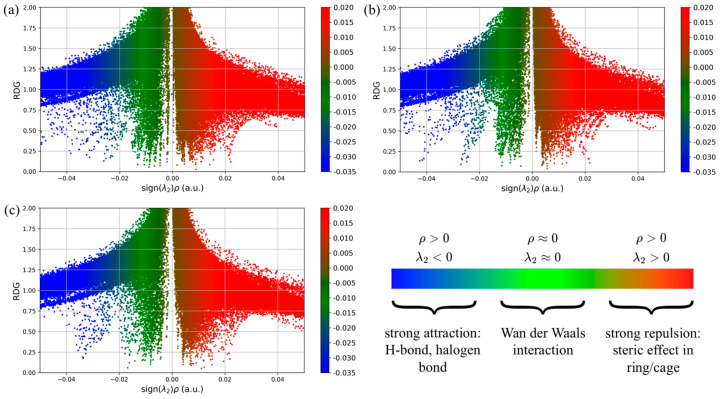
RDG scattering diagram of the intermolecular system of nadolol and (**a**) citric, (**b**) oxalic, and (**c**) acetic acid.

A detailed overview of the non-covalent interactions between nadolol and the observed organic acids can be obtained by analyzing the RDG value in relation to the (λ_2_)ρ value, which can be seen in Figure 6. By evaluating the RDG scatter diagram, the type of interaction can be identified based on the sign(λ_2_)ρ value. If the value of λ_2_ < 0 and ρ > 0 (blue points), there is a strong attractive interaction, i.e., the formation of a hydrogen bond in the investigated system. When the values of λ_2_ and ρ are approximately equal to zero (green dots), weak Van der Waals interactions are detected, and the bond is weaker compared to the previously mentioned one. The red dots represent strong repulsion, indicating that the values of λ_2_ and ρ are positive.

**Figure 7:**
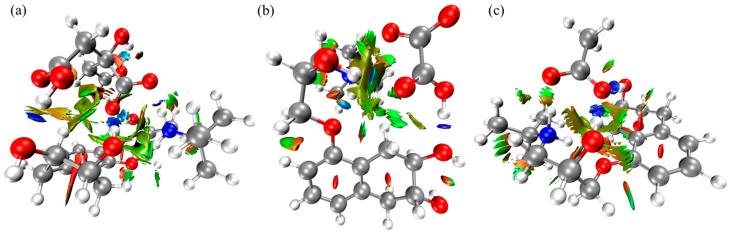
RDG surface of the intermolecular system of nadolol and (**a**) citric, (**b**) oxalic, and (**c**) acetic acid. Atom color coding: carbon—grey, oxygen—red, nitrogen—blue and hydrogen—white.

A more significant number of blue dots can be observed in the case of the nadolol/citric acid system (Figure 6) compared to the other observed organic acids, indicating stronger binding by hydrogen bonds. Red dots are approximately equally represented in all three systems. To give a more detailed view, RDG surfaces of the observed intermolecular systems are mapped according to the intensity of non-covalent interactions (Figure 7).

Figure 7a reveals intense surfaces between the hydroxyl groups of nadolol’s ring and side chain and the hydrogen atoms of citric acid. Strong interactions are also evident between the amino group of nadolol and the carboxyl group of citric acid. A total of four H-bonds makes the nadolol/citric acid system the most stable compared to the other two observed systems with two H-bonds, which accounts for the lowest degradation efficiency of nadolol in the presence of citric acid (Figure 2b).

The identified interaction patterns are highly relevant for heteroatom-containing PACs interacting with oxide surfaces under environmentally realistic conditions.

### 2.4. UV Spectrophotometric Behavior

**Figure 8:**
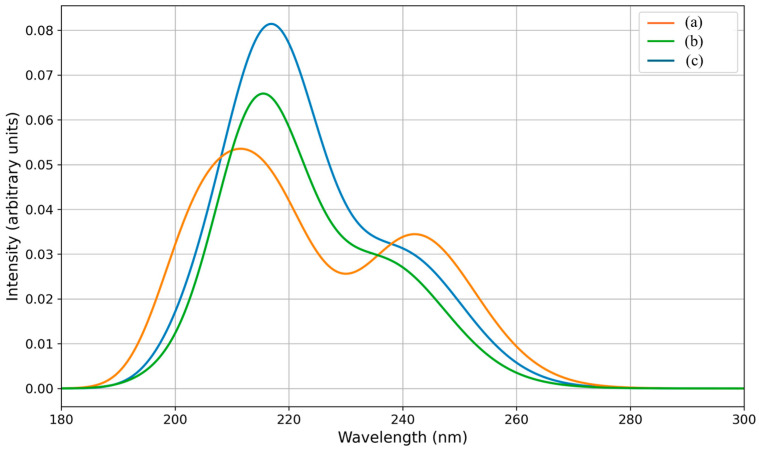
UV absorption spectra of (a) nadolol/citric acid, (b) nadolol/oxalic acid, and (c) nadolol/acetic acid systems.

Using DFT and time-dependent DFT (TD-DFT), we simulated the UV absorption spectra of nadolol in the presence of citric, oxalic, and acetic acids (Figure 8). With these calculations, we aimed to better understand the changes in nadolol’s electronic structure and optical transitions resulting from these interactions. Nadolol contains a basic amine group (Figure 1), which can be protonated in the presence of citric, oxalic, and acetic acids. This protonation alters the electron distribution in the molecule, thereby affecting its electronic transitions and leading to changes in absorbance intensity [37]. Results indicate that at approximately 215 nm, the absorption maximum of the nadolol/acetic acid system is the highest compared to the other two observed systems. Experiments indicate that acetic acid has the least impact on reducing the efficiency of nadolol’s degradation (Figure 2b), which can be explained by its least effect on the photoexcitation of nadolol (Figure 8).

The system with citric acid absorbs the least amount of radiation at approximately 215 nm. The UV absorption spectra of all three systems exhibit a peak at around 240 nm, with slightly higher intensity for the citric acid system. However, its absorption at 240 nm remains lower than the absorption of the other two systems at 215 nm, which corresponds to the lowest degradation efficiency of nadolol in the presence of citric acid.

Since the photocatalytic process is initiated by photoexcitation of the organic molecule, reduced absorption intensity at the relevant excitation wavelength implies less efficient population of excited electronic states and consequently lower degradation efficiency.

## 3. Materials and Methods

### 3.1. Chemicals and Solutions

Chemicals used were nadolol ((2R,3S)-5-[3-(tert-butylamino)-2-hydroxypropoxy]-1,2,3,4-tetrahydronaphthalene-2,3-diol, C_17_H_27_NO_4_, M = 309.4 g/mol, ≥99% purity, Sigma-Aldrich, Steinheim, Germany), TiO_2_ (100% anatase, Sigma-Aldrich, specific surface area 35–65 m^2^/g, particle size 20 nm, Sigma-Aldrich, Steinheim, Germany), citric acid (C_6_H_8_O_7_, M = 192.12 g/mol, ≥99% purity, Sigma-Aldrich, Germany), oxalic acid (C_2_H_2_O_4_, M = 90.04 g/mol, ≥99% purity, Centrohem, Stara Pazova, Serbia), acetic acid (CH_3_COOH, M = 60.05 g/mol, ≥99% purity, Polskie Odczynniki Chemiczne S.A., Gliwice, Poland), perchloric acid (HClO_4_, M = 100.46 g/mol, ≥99% purity, Sigma-Aldrich, Germany), acetonitrile (ACN, C_2_H_3_N, M = 41.05 g/mol, ≥99% purity, Sigma-Aldrich, Germany), and orthophosphoric acid (H_3_PO_4_, M = 97.99 g/mol, 85% purity, Lachema, Brno, Czech Republic). The chemicals were of reagent grade and were used without further purification.

The stock nadolol solutions were made using UPW (κ = 0.055 μS/cm, pH 6.6). The solutions were stored in a light-free environment at a temperature of 4 °C.

### 3.2. Photodegradation Experiments

**Figure 9:**
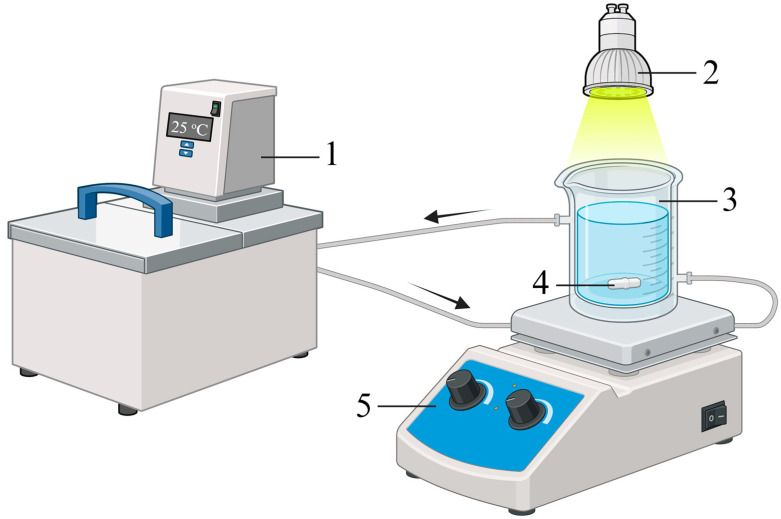
Schematic illustration of the photoreactor: (1) circulation thermostat, (2) UV-LED radiation source, (3) double glass photochemical cell, (4) magnet, and (5) magnetic stirrer.

The photocatalytic degradation of nadolol was carried out using a double glass photochemical cell onto which the light beam was focused (Figure 9) [38]. The total volume of the cell was approximately 250 cm^3^. Experiments were performed using 60 cm^3^ of 0.05 mmol/dm^3^ nadolol solution. In the photocatalytic degradation experiments, the solution contained 1.0 mg/cm^3^ of TiO_2_ and 3.00 mmol/dm^3^ of citric, oxalic, or acetic acids. Before irradiation, the reaction mixture was sonicated for 15 min in the dark to establish adsorption/desorption equilibrium. The reaction mixtures were exposed to UV-LED irradiation for 120 min using a 5 W UV-LED Lamp (Enjoydeal, Shenzhen, China, type: MR16 AC 85-265V/12, with an intensity of 107.23 W/m^2^ in the visible region, and 14.356 W/m^2^ in the UV region), with the source positioned 50 mm from the photochemical cell. Before irradiation, the reaction mixture was maintained at a constant temperature of 25 °C using a water circulation thermostat. The solution was stirred with a magnetic stirring bar throughout the experiments.

### 3.3. Analytical Procedure

Samples of the reaction mixture were collected before and during cell exposure to the UV-LED irradiation. Then, the samples were filtered through a Millipore membrane filter (Burlington, MA, USA, Milex-GV, 0.22 µm) and collected in a chromatographic vial.

To study degradation kinetics, 20 µL of the filtrate was analyzed by Shimadzu UFLC-PDA (Nakagyo-ku, Japan, Eclipse XDB-C18 column, 150 mm × 4.6 mm i.d., particle size 5 µm, 30 °C) [7].

For control experiments, the pH was adjusted using perchloric acid (HClO_4_) to match the values observed in the presence of organic acids.

Radiation energy flux was measured using a Delta Ohm HD 2102.2 radiometer (Padova, Italy) equipped with LP 471 UV (315–400 nm) and LP 471 RAD (400–1050 nm) sensors. During the degradation process, a combination glass electrode (pH-Electrode SenTix 20, WTW) connected to a pH meter (pH/Cond 340i, WTW) was used to track the pH values.

### 3.4. Computational Details

The most stable conformation of the system nadolol/organic acid was obtained using the GOAT procedure implemented in the ORCA 6 package [39,40,41,42,43] based on the GFN2-xTB semi-empirical method [44,45,46]. Later, the intermolecular systems were reoptimized using the B3LYP functional [47,48,49,50], empirically corrected for dispersion interactions (B3LYP-D3) [51,52,53], using the Jaguar program [54,55,56,57,58] as implemented in the Schrödinger Materials Science Suite 2022-2. Binding energies between nadolol and selected acids were obtained by the standard equation, using B3LYP-D3 functional [36,37,38,39], with 6-31G(d,p) basis set. The selected level of theory was chosen to provide a reasonable balance between computational efficiency and accuracy for evaluating relative binding trends in the studied systems. The basis set superposition error was treated with the standard method introduced by Boys and Bernardi [59].

The ORCA 6 program was used to perform single-point DFT energy calculations on the optimized structures [39,40,41,42,43]. This process generated the files necessary to create RDG plots and RDG surfaces using the Multiwfn program [60] based on the approach described by Johnson et al. [61]. In addition, the online RDG calculator is available at atomistica.online, the molecular modeling platform (accessed freely at https://atomistica.online) [62,63,64,65], which uses the Multiwfn program in the background to generate RDG scatterplots and .cub files, containing volumetric data and atomic-structure information, for RDG surfaces, was also used in the present study. The resulting .cub files were used to generate RDG surfaces using the VMD program [66,67,68,69,70,71,72,73].

To simulate the UV-Vis absorption spectra of nadolol in the presence of citric, oxalic, and acetic acids, electronic excitations were calculated using time-dependent density functional theory (TD-DFT). The calculations were performed with the CAM-B3LYP functional [74] in combination with the 6-311++G(d,p) basis set, using ORCA 6.1. program. In each case, 40 excited states were requested. The simulated UV-Vis spectra were constructed by applying Gaussian broadening to the calculated excitation energies and oscillator strengths, considering only transitions with wavelengths longer than 200 nm. Solvent effects were taken into account via the CPCM model [75].

In the periodic DFT calculations, the PBE functional empirically corrected for dispersion forces (PBE-D3) was used [76,77], in combination with GBRV pseudopotentials [78]. Literature data indicate that the (101) surface is the most stable and representative crystal direction of TiO_2_. Therefore, the TiO_2_ (101) surface model with two layers was utilized in the simulations.

First, the pure TiO_2_ bilayer surface was geometrically optimized without any constraints on atomic positions or cell parameters. Then the acid molecules were adsorbed on the surface, after which the geometric optimization was repeated with fixed cell dimensions, allowing optimization of only atomic positions. The limit values of kinetic energy are set at 40 Ry for wave functions and 200 Ry for charge density. A 2 × 2 × 1 grid was used to sample the Brillouin zone. Geometry optimizations were performed using the BFGS quasi-Newtonian algorithm, with convergence criteria set to 1 × 10^−6^ Ry for SCF cycles, 1 × 10^−6^ Ry for total energy change, and 0.001 Ry/Bohr for atomic forces. All periodic DFT calculations were performed using Quantum ESPRESSO v7.2 [79,80,81,82].

## 4. Conclusions

Results demonstrated that while TiO_2_ exhibits high photocatalytic efficiency for nadolol degradation under UV-LED irradiation in ultrapure water, the presence of naturally occurring organic acids can substantially inhibit the process. Citric, oxalic, and acetic acids were shown to reduce degradation efficiency to different extents, with citric acid exerting the strongest inhibitory effect.

Combined experimental observations and molecular- and periodic-level DFT calculations revealed that this inhibition cannot be attributed solely to pH effects, but rather arises from specific intermolecular interactions and competitive adsorption phenomena. Strong binding of organic acids to both nadolol and the TiO_2_ surface leads to partial blocking of photocatalytically active sites, thereby limiting pollutant degradation. Among the investigated acids, citric acid forms the most stable intermolecular systems with both nadolol and TiO_2_, as confirmed by binding energy calculations and RDG analysis, explaining its pronounced inhibitory role. Acetic acid exhibits weaker interactions and minimal impact on nadolol photoexcitation, resulting in the least inhibition of photocatalytic activity.

The results highlight the critical influence of low-molecular-weight organic acids on TiO_2_-based photocatalytic systems and emphasize the necessity of considering real-water matrix components when evaluating and designing photocatalytic water treatment processes. Although this study focuses on an aromatic pharmaceutical, the identified inhibition mechanisms are directly transferable to polycyclic aromatic compounds (PACs), whose environmental persistence is likewise governed by competitive adsorption and noncovalent surface interactions. The integrated experimental–computational approach adopted in this work provides a robust framework for understanding inhibition mechanisms in complex aqueous environments and can support the rational development of more efficient and environmentally relevant photocatalytic technologies.

The mechanistic insights obtained from single-acid systems provide a basis for future studies involving complex wastewater matrices.

## Figures and Tables

**Table 1:** Comparing the photocatalytic performance of different TiO_2_-based materials for the degradation of beta-blockers after 30 min of irradiation.

Photocatalyst	Beta-Blocker	Degradation Efficiency (%)	Reference
Immobilized TiO_2_	Nadolol	83	[33]
TiO_2_ Degussa P-25	Metoprolol	2	[34]
Biochar-Doped TiO_2_	Propranolol	56	[35]
TiO_2_/Zeolite	Atenolol	95	[36]
TiO_2_ anatase	Nadolol	88	Our study

**Table 2:** Binding energy [kcal/mol] between nadolol or TiO_2_ and organic acids.

Organic Acid	Citric Acid	Oxalic Acid	Acetic Acid
nadolol	−29.13	−26.01	−23.10
TiO_2_	−42.04	−21.02	−15.37

## Data Availability

The original contributions presented in this study are included in the article. Further inquiries can be directed to the corresponding author(s).

## References

[1] Li L., Chen C., Li D., Breivik K., Abbasi G., Li Y.-F. (2023). What Do We Know about the Production and Release of Persistent Organic Pollutants in the Global Environment?. Environ. Sci. Adv..

[2] Liu J., Qi W., Xu M., Thomas T., Liu S., Yang M. (2023). Piezocatalytic Techniques in Environmental Remediation. Angew. Chem..

[3] Saravanan A., Kumar P.S., Jeevanantham S., Anubha M., Jayashree S. (2022). Degradation of Toxic Agrochemicals and Pharmaceutical Pollutants: Effective and Alternative Approaches toward Photocatalysis. Environ. Pollut..

[4] Petruzzelli G., Pezzarossa B., Pedron F. (2025). The Fate of Chemical Contaminants in Soil with a View to Potential Risk to Human Health: A Review. Environments.

[5] Hamza M., Ayinla R.T., Elsayed I., Hassan E.B. (2025). Understanding PFAS Adsorption: How Molecular Structure Affects Sustainable Water Treatment. Environments.

[6] Ye Z., Guo Z., Wang J., Zhang L., Guo Y., Yoshimura C., Niu J. (2022). Photodegradation of Acebutolol in Natural Waters: Important Roles of Carbonate Radical and Hydroxyl Radical. Chemosphere.

[7] Bilić A., Savanović M.M., Armaković S., Četojević-Simin D.D., Srđenović Čonić B., Kladar N., Armaković S.J. (2023). Exploring the Influence of Free Radicals on Photolytic Removal of Nadolol from Water: Mechanism of Degradation and Toxicity of Intermediates. Front. Environ. Sci..

[8] Lucia C., Laudicina V.A., Badalucco L., Galati A., Palazzolo E., Torregrossa M., Viviani G., Corsino S.F. (2022). Challenges and Opportunities for Citrus Wastewater Management and Valorisation: A Review. J. Environ. Manag..

[9] Pampinella D., Laudicina V.A., Saiano F., Palazzolo E., Badalucco L., Ioppolo A. (2024). Dynamics of Water-Soluble Metals in Soil Moistened with Citrus Wastewaters Depends on Soil Reaction and Organic Acids. Water.

[10] Tran H.D., Quan Nguyen D., Do P.T., Tran U.N.P. (2023). Kinetics of Photocatalytic Degradation of Organic Compounds: A Mini-Review and New Approach. RSC Adv..

[11] Armaković S.J., Savanović M.M., Armaković S. (2023). Titanium Dioxide as the Most Used Photocatalyst for Water Purification: An Overview. Catalysts.

[12] Parameswari M., Jayamoorthy K. (2025). A Comprehensive Overview of Titanium Dioxide for Sensor and Medicinal Applications. Microchem. J..

[13] Nagaraj K., Radha S., Deepa C.G., Raja K., Umapathy V., Badgujar N.P., Parekh N.M., Manimegalai T., Archana Devi L., Uthra C. (2025). Photocatalytic Advancements and Applications of Titanium Dioxide (TiO_2_): Progress in Biomedical, Environmental, and Energy Sustainability. Next Res..

[14] Impemba S., Provinciali G., Filippi J., Caporali S., Muzzi B., Casini A., Caporali M. (2024). Tightly Interfaced Cu_2_O with In_2_O_3_ to Promote Hydrogen Evolution in Presence of Biomass-Derived Alcohols. ChemNanoMat.

[15] Jones K.C. (2021). Persistent Organic Pollutants (POPs) and Related Chemicals in the Global Environment: Some Personal Reflections. Environ. Sci. Technol..

[16] Zheng F., Queirós J.M., Martins P.M., De Luis R.F., Fidalgo-Marijuan A., Vilas-Vilela J.L., Lanceros-Méndez S., Reguera J. (2023). Au-Sensitised TiO_2_ and ZnO Nanoparticles for Broadband Pharmaceuticals Photocatalytic Degradation in Water Remediation. Colloids Surf. A Physicochem. Eng. Asp..

[17] Gnanaprakasam A., Sivakumar V.M., Sivayogavalli P.L., Thirumarimurugan M. (2015). Characterization of TiO_2_ and ZnO Nanoparticles and Their Applications in Photocatalytic Degradation of Azodyes. Ecotoxicol. Environ. Saf..

[18] Topalov B., Savanović M. (2025). Photocatalytic Properties and Application of TiO_2_ and ZnO Nanoparticles. AIDASCO Rev..

[19] Tomić J., Malinović N. (2023). Titanium Dioxide Photocatalyst: Present Situation and Future Approaches. AIDASCO Rev..

[20] Comparelli R., Fanizza E., Curri M.L., Cozzoli P.D., Mascolo G., Passino R., Agostiano A. (2005). Photocatalytic Degradation of Azo Dyes by Organic-Capped Anatase TiO_2_ Nanocrystals Immobilized onto Substrates. Appl. Catal. B Environ..

[21] Comparelli R., Fanizza E., Curri M.L., Cozzoli P.D., Mascolo G., Agostiano A. (2005). UV-Induced Photocatalytic Degradation of Azo Dyes by Organic-Capped ZnO Nanocrystals Immobilized onto Substrates. Appl. Catal. B Environ..

[22] Kaya S., Banerjee P., Saha S.K., Tüzün B., Kaya C. (2016). Theoretical Evaluation of Some Benzotriazole and Phospono Derivatives as Aluminum Corrosion Inhibitors: DFT and Molecular Dynamics Simulation Approaches. RSC Adv..

[23] Al-Otaibi J.S., Mary Y.S., Mary Y.S., Kaya S., Serdaroglu G. (2022). DFT Computational Study of Trihalogenated Aniline Derivative’s Adsorption onto Graphene/Fullerene/Fullerene-like Nanocages, X12Y12 (X = Al, B, and Y = N, P). J. Biomol. Struct. Dyn..

[24] Kaya S., Lgaz H., Thakkur A., Kumar A., Özbakır Işın D., Karakuş N., Ben Ahmed S. (2023). Molecular Insights into the Corrosion Inhibition Mechanism of Omeprazole and Tinidazole: A Theoretical Investigation. Mol. Simul..

[25] Thomas R., Mary Y.S., Resmi K.S., Narayana B., Sarojini S.B.K., Armaković S., Armaković S.J., Vijayakumar G., Alsenoy C.V., Mohan B.J. (2019). Synthesis and Spectroscopic Study of Two New Pyrazole Derivatives with Detailed Computational Evaluation of Their Reactivity and Pharmaceutical Potential. J. Mol. Struct..

[26] Beegum S., Mary Y.S., Mary Y.S., Thomas R., Armaković S., Armaković S.J., Zitko J., Dolezal M., Van Alsenoy C. (2020). Exploring the Detailed Spectroscopic Characteristics, Chemical and Biological Activity of Two Cyanopyrazine-2-Carboxamide Derivatives Using Experimental and Theoretical Tools. Spectrochim. Acta Part A Mol. Biomol. Spectrosc..

[27] Al-Otaibi J.S., Mary Y.S., Armaković S., Thomas R. (2020). Hybrid and Bioactive Cocrystals of Pyrazinamide with Hydroxybenzoic Acids: Detailed Study of Structure, Spectroscopic Characteristics, Other Potential Applications and Noncovalent Interactions Using SAPT. J. Mol. Struct..

[28] Thomas R. (2025). A Systematic Computational Protocol for Deconstructing Non-Covalent Interactions: BerchNCI 1.0. AIDASCO Rev..

[29] Sulay R., Sunny S.A., Bushramol S., Arshana A.N., Alzahrani A.Y.A., Thomas R. (2026). The Hydration Blueprint of Polylactic Acid: Computational Decoding of Noncovalent Interactions for Predictive Biodegradation. J. Comput. Chem..

[30] Sulay R., Sunny S.A., Alzahrani A.Y.A., Thomas R. (2025). Unraveling the Weak Yet Vital: A High-Level DFT Exploration of Non-Covalent Interactions in Hydrated Polyethylene Glycol and Methoxy Polyethylene Glycol Systems. Adv. Theory Sims.

[31] Sunny S.A., Rajan R.F., Thilakan S., Thomas F., Pooventhiran T., Alzahrani A.Y.A., Thomas R. (2026). Investigating the Role of Noncovalent Interactions in Vadadustat’s Solubility and Stability with Selected Solvents. ChemistrySelect.

[32] Kweinor Tetteh E., Obotey Ezugbe E., Asante-Sackey D., Armah E.K., Rathilal S. (2021). Response Surface Methodology: Photocatalytic Degradation Kinetics of Basic Blue 41 Dye Using Activated Carbon with TiO_2_. Molecules.

[33] Píšťková V., Tasbihi M., Vávrová M., Štangar U.L. (2015). Photocatalytic Degradation of β-Blockers by Using Immobilized Titania/Silica on Glass Slides. J. Photochem. Photobiol. A Chem..

[34] Romero V., Marco P., Giménez J., Esplugas S. (2013). Adsorption and Photocatalytic Decomposition of the β-Blocker Metoprolol in Aqueous Titanium Dioxide Suspensions: Kinetics, Intermediates, and Degradation Pathways. Int. J. Photoenergy.

[35] Kowalczyk A., Zgardzińska B., Osipiuk K., Jędruchniewicz K., Tyszczuk-Rotko K., Goździuk M., Wang H., Czech B. (2023). The Visible-Light-Driven Activity of Biochar-Doped TiO_2_ Photocatalysts in β-Blockers Removal from Water. Materials.

[36] Sarabyar S., Farahbakhsh A., Tahmasebi H.A., Mahmoodzadeh Vaziri B., Khosroyar S. (2024). Enhancing Photocatalytic Degradation of Beta-Blocker Drugs Using TiO_2_ NPs/Zeolite and ZnO NPs/Zeolite as Photocatalysts: Optimization and Kinetic Investigations. Sci. Rep..

[37] Grante I., Actins A., Orola L. (2014). Protonation Effects on the UV/Vis Absorption Spectra of Imatinib: A Theoretical and Experimental Study. Spectrochim. Acta Part A Mol. Biomol. Spectrosc..

[38] BioRender. https://BioRender.com.

[39] Neese F. (2022). Software Update: The ORCA Program System, Version 5.0. WIRES Comput. Mol. Sci..

[40] Neese F. (2018). Software Update: The ORCA Program System, Version 4.0. WIRES Comput. Mol. Sci..

[41] Neese F. (2012). The ORCA Program System. WIRES Comput. Mol. Sci..

[42] Neese F., Wennmohs F., Becker U., Riplinger C. (2020). The ORCA Quantum Chemistry Program Package. J. Chem. Phys..

[43] Neese F. (2022). The SHARK Integral Generation and Digestion System. J. Comp. Chem..

[44] Bannwarth C., Caldeweyher E., Ehlert S., Hansen A., Pracht P., Seibert J., Spicher S., Grimme S. (2021). Extended Tight-Binding Quantum Chemistry Methods. WIREs Comput. Mol. Sci..

[45] Bannwarth C., Ehlert S., Grimme S. (2019). GFN2-xTB—An Accurate and Broadly Parametrized Self-Consistent Tight-Binding Quantum Chemical Method with Multipole Electrostatics and Density-Dependent Dispersion Contributions. J. Chem. Theory Comput..

[46] Grimme S., Bannwarth C., Shushkov P. (2017). A Robust and Accurate Tight-Binding Quantum Chemical Method for Structures, Vibrational Frequencies, and Noncovalent Interactions of Large Molecular Systems Parametrized for All Spd-Block Elements (Z = 1–86). J. Chem. Theory Comput..

[47] Stephens P.J., Devlin F.J., Chabalowski C.F., Frisch M.J. (1994). Ab Initio Calculation of Vibrational Absorption and Circular Dichroism Spectra Using Density Functional Force Fields. J. Phys. Chem..

[48] Vosko S.H., Wilk L., Nusair M. (1980). Accurate Spin-Dependent Electron Liquid Correlation Energies for Local Spin Density Calculations: A Critical Analysis. Can. J. Phys..

[49] Lee C., Yang W., Parr R.G. (1988). Development of the Colle-Salvetti Correlation-Energy Formula into a Functional of the Electron Density. Phys. Rev. B.

[50] Becke A.D. (1993). Density-functional Thermochemistry. III. The Role of Exact Exchange. J. Chem. Phys..

[51] Smith D.G.A., Burns L.A., Patkowski K., Sherrill C.D. (2016). Revised Damping Parameters for the D3 Dispersion Correction to Density Functional Theory. J. Phys. Chem. Lett..

[52] Grimme S., Ehrlich S., Goerigk L. (2011). Effect of the Damping Function in Dispersion Corrected Density Functional Theory. J. Comput. Chem..

[53] Grimme S., Antony J., Ehrlich S., Krieg H. (2010). A Consistent and Accurate Ab Initio Parametrization of Density Functional Dispersion Correction (DFT-D) for the 94 Elements H-Pu. J. Chem. Phys..

[54] Jacobson L.D., Bochevarov A.D., Watson M.A., Hughes T.F., Rinaldo D., Ehrlich S., Steinbrecher T.B., Vaitheeswaran S., Philipp D.M., Halls M.D. (2017). Automated Transition State Search and Its Application to Diverse Types of Organic Reactions. J. Chem. Theory Comput..

[55] Cao Y., Hughes T., Giesen D., Halls M.D., Goldberg A., Vadicherla T.R., Sastry M., Patel B., Sherman W., Weisman A.L. (2016). Highly Efficient Implementation of Pseudospectral Time-Dependent Density-Functional Theory for the Calculation of Excitation Energies of Large Molecules. J. Comput. Chem..

[56] Bochevarov A.D., Harder E., Hughes T.F., Greenwood J.R., Braden D.A., Philipp D.M., Rinaldo D., Halls M.D., Zhang J., Friesner R.A. (2013). Jaguar: A High-Performance Quantum Chemistry Software Program with Strengths in Life and Materials Sciences. Int. J. Quantum Chem..

[57] Cao Y., Halls M.D., Vadicherla T.R., Friesner R.A. (2021). Pseudospectral Implementations of Long-Range Corrected Density Functional Theory. J. Comput. Chem..

[58] Cao Y., Balduf T., Beachy M.D., Bennett M.C., Bochevarov A.D., Chien A., Dub P.A., Dyall K.G., Furness J.W., Halls M.D. (2024). Quantum Chemical Package Jaguar: A Survey of Recent Developments and Unique Features. J. Chem. Phys..

[59] Boys S.F., Bernardi F. (1970). The Calculation of Small Molecular Interactions by the Differences of Separate Total Energies. Some Procedures with Reduced Errors. Mol. Phys..

[60] Lu T., Chen F. (2012). Multiwfn: A Multifunctional Wavefunction Analyzer. J. Comput. Chem..

[61] Johnson E.R., Keinan S., Mori-Sánchez P., Contreras-García J., Cohen A.J., Yang W. (2010). Revealing Noncovalent Interactions. J. Am. Chem. Soc..

[62] Armaković S., Armaković S.J. (2025). Predicting Properties of Imidazolium-Based Ionic Liquids via Atomistica Online: Machine Learning Models and Web Tools. Computation.

[63] Armaković S., Armaković S.J. (2024). Online and Desktop Graphical User Interfaces for Xtb Programme from Atomistica.Online Platform. Mol. Simul..

[64] Armaković S., Armaković S.J. (2023). Atomistica.Online—Web Application for Generating Input Files for ORCA Molecular Modelling Package Made with the Anvil Platform. Mol. Simul..

[65] Armaković S., Armaković S. (2025). Recent Developments in Atomistic Modeling: Machine Learning Models and Datasets, Methods, Software Releases, and Scientific Events. AIDASCO Rev..

[66] Humphrey W., Dalke A., Schulten K. (1996). VMD—Visual Molecular Dynamics. J. Mol. Graph..

[67] Eargle J., Wright D., Luthey-Schulten Z. (2006). Multiple Alignment of Protein Structures and Sequences for VMD. Bioinformatics.

[68] Stone J., Gullingsrud J., Grayson P., Schulten K., Hughes J.F., Séquin C.H. (2001). A System for Interactive Molecular Dynamics Simulation. Proceedings of the 2001 ACM Symposium on Interactive 3D Graphics.

[69] Frishman D., Argos P. (1995). Knowledge-Based Secondary Structure Assignment. Proteins Struct. Funct. Genet..

[70] Sanner M., Olsen A., Spehner J.-C. (1995). Fast and Robust Computation of Molecular Surfaces. Proceedings of the 11th ACM Symposium on Computational Geometry.

[71] Stone J.E. (1998). An Efficient Library for Parallel Ray Tracing and Animation. Master’s Thesis.

[72] Varshney A., Brooks F.P., Wright W.V. (1994). Linearly Scalable Computation of Smooth Molecular Surfaces. IEEE Comput. Graph. Appl..

[73] Levine B.G., Stone J.E., Kohlmeyer A. (2011). Fast Analysis of Molecular Dynamics Trajectories with Graphics Processing Units—Radial Distribution Function Histogramming. J. Comput. Phys..

[74] Yanai T., Tew D.P., Handy N.C. (2004). A New Hybrid Exchange–Correlation Functional Using the Coulomb-Attenuating Method (CAM-B3LYP). Chem. Phys. Lett..

[75] Barone V., Cossi M. (1998). Quantum Calculation of Molecular Energies and Energy Gradients in Solution by a Conductor Solvent Model. J. Phys. Chem. A.

[76] Perdew J.P., Burke K., Ernzerhof M. (1996). Generalized Gradient Approximation Made Simple. Phys. Rev. Lett..

[77] Perdew J.P., Burke K., Ernzerhof M. (1997). Generalized Gradient Approximation Made Simple [Phys. Rev. Lett. 77, 3865 (1996)]. Phys. Rev. Lett..

[78] Garrity K.F., Bennett J.W., Rabe K.M., Vanderbilt D. (2014). Pseudopotentials for High-Throughput DFT Calculations. Comput. Mater. Sci..

[79] Scandolo S., Giannozzi P., Cavazzoni C., de Gironcoli S., Pasquarello A., Baroni S. (2005). First-Principles Codes for Computational Crystallography in the Quantum-ESPRESSO Package. Z. Krist.-Cryst. Mater..

[80] Giannozzi P., Baseggio O., Bonfà P., Brunato D., Car R., Carnimeo I., Cavazzoni C., de Gironcoli S., Delugas P., Ferrari Ruffino F. (2020). Quantum ESPRESSO toward the Exascale. J. Chem. Phys..

[81] Giannozzi P., Andreussi O., Brumme T., Bunau O., Buongiorno Nardelli M., Calandra M., Car R., Cavazzoni C., Ceresoli D., Cococcioni M. (2017). Advanced Capabilities for Materials Modelling with Quantum ESPRESSO. J. Phys. Condens. Matter.

[82] Giannozzi P., Baroni S., Bonini N., Calandra M., Car R., Cavazzoni C., Ceresoli D., Chiarotti G.L., Cococcioni M., Dabo I. (2009). QUANTUM ESPRESSO: A Modular and Open-Source Software Project for Quantum Simulations of Materials. J. Phys. Condens. Matter.

